# Henoch-Schonlein Purpura Presenting as Upper Gastrointestinal Bleed in an Adult Patient

**DOI:** 10.7759/cureus.13879

**Published:** 2021-03-14

**Authors:** Yasmin Khader, Cameron Burmeister, Dipen Patel, Amala Ambati, Nezam Altorok

**Affiliations:** 1 Internal Medicine, University of Toledo, Toledo, USA; 2 Rheumatology, University of Toledo, Toledo, USA

**Keywords:** immunoglobulin a vasculitis, henoch-schonlein purpura, upper gastrointestinal bleed

## Abstract

Henoch-Schonlein purpura (HSP) is a multi-system autoimmune disease that is relatively common in pediatric patients. HSP usually manifests as palpable purpura, arthralgia, abdominal pain, and acute kidney injury. Here, we present a case of an adult male with hematemesis as the initial presenting symptom of HSP.

A previously healthy, 18-year-old Caucasian male presented with a one-day history of hematemesis associated with abdominal pain and non-bloody diarrhea. He also reported bilateral knee and ankle arthralgias with a painless skin rash on both lower extremities. Physical exam was positive for palpable, purpuric, non-blanchable skin rash involving bilateral lower extremities. Notable labs on admission included a white cell count of 10.8 x 10^9^/L and C-reactive protein of 4.8 mg/L. Upper endoscopy showed non-bleeding erosive gastropathy and duodenal erosions. Skin biopsy of the left leg showed immunoglobulin A (IgA) deposition within the walls of the superficial dermal vessels. The patient was started on intravenous methylprednisolone 500 mg daily followed by a steroid taper. Due to incomplete clinical response to steroids, mycophenolate mofetil 1000 mg twice daily was added and maintained for three months. His symptoms improved significantly, and he no longer complained of abdominal pain or diarrhea.

Gastrointestinal manifestations are common in HSP patients. However, the diagnosis will be challenging when these symptoms precede other classical manifestations of HSP. History and physical exam are key components in accurately diagnosing HSP; nevertheless, skin biopsy remains the gold standard to confirm the diagnosis.

## Introduction

Immunoglobulin A (IgA) vasculitis, also known as Henoch-Schonlein purpura (HSP), is a systemic autoimmune vasculitis that affects the small blood vessels [1]. It commonly presents with a tetrad of symptoms, including palpable purpura, arthralgia, abdominal pain, and acute kidney injury [2]. Rarely, life-threatening gastrointestinal (GI) complications can occur, which include intussusception, bowel necrosis, bowel perforation, and GI bleeding [3]. Here, we present a case of an adult male with hematemesis as the initial presenting symptom of IgA vasculitis.

## Case presentation

A previously healthy 18-year-old Caucasian male presented with a one-day history of bloody vomitus associated with fever, chills, abdominal pain, and non-bloody diarrhea. His abdominal pain was centrally located, constant, dull-aching, non-radiating, and 6/10 in severity. He also reported rhinorrhea and sore throat two weeks prior to presentation. One week after the onset of symptoms, the patient noticed bilateral knee and ankle joint pain, which limited weight-bearing and ambulation. These symptoms were followed by a painless, purpuric skin rash on the left ankle that extended proximally and bilaterally. The patient denied any previous similar episodes, chest pain, shortness of breath, cough, dysphagia, odynophagia, nausea, or vomiting. Family history was negative for similar conditions. The patient denied smoking, alcohol, or illicit drug use.

**Figure 1:**
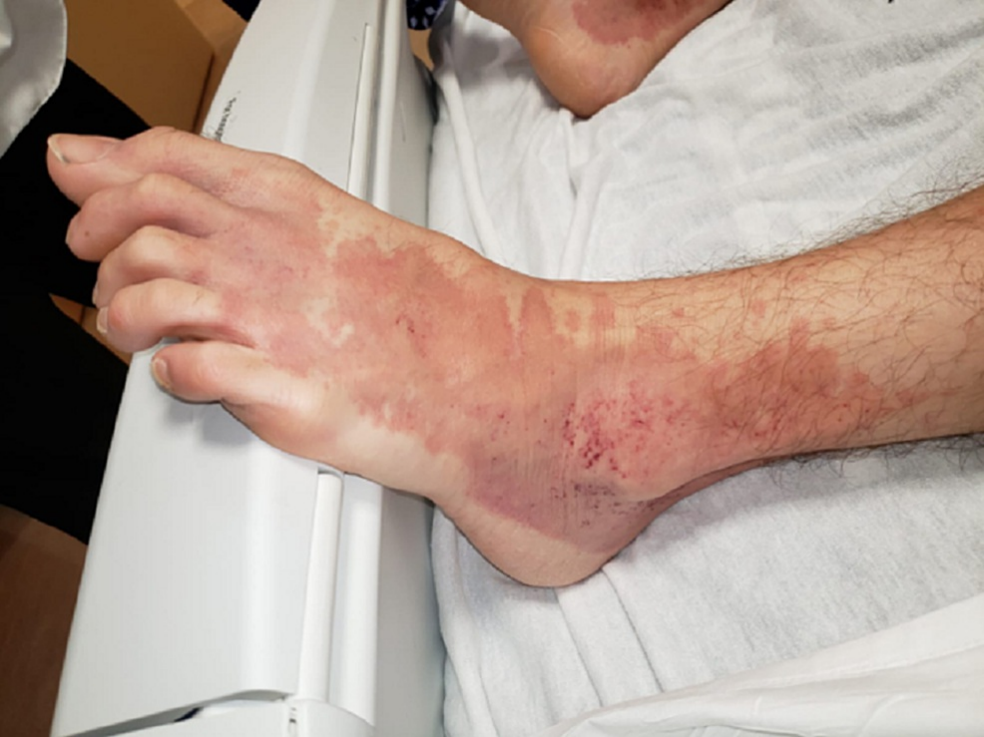
Palpable purpura on the lower extremity

In the emergency department, the patient was afebrile and hemodynamically stable with a temperature of 36.9 °C (98.4 °F), heart rate of 67 beats per minute, respiratory rate of 16 breaths per minute, blood pressure of 147/73 mmHg, and oxygen saturation (SpO2) of 99% on room air. The head and neck exam showed oropharyngeal erythema with petechiae on the hard palate and uvula, as well as anterior cervical lymphadenopathy. The cardiopulmonary exam was within normal limits. The abdomen was soft and non-distended with normal bowel sounds; no masses or organomegaly were noted. The musculoskeletal exam was negative for joint swelling, erythema, or tenderness. Skin exam was significant for a palpable, purpuric, non-blanchable skin rash involving bilateral lower extremities (Figure 1).

Notable labs on admission included the following: white blood count of 10.8 x 10^9^/L, hemoglobin of 15.8 g/dl, platelets of 243 x 10^9^/L, creatinine of 0.97 mg/dl, aspartate aminotransferase of 47 units/L, alanine aminotransferase of 58 units/L, and C-reactive protein of 4.8 mg/L. Urine dipstick was positive for trace hemoglobin and +2 proteinuria. The following labs were within normal limits: total bilirubin, alkaline phosphatase, antinuclear antibodies, antineutrophil cytoplasmic antibodies, C3, C4, human immunodeficiency virus screen, hepatitis panel, prothrombin time (PT), activated partial thromboplastin time (aPTT), and blood cultures. The chest X-ray, abdomen ultrasound, and abdomen/pelvis computed tomography (CT) scan were unremarkable.

**Figure 2:**
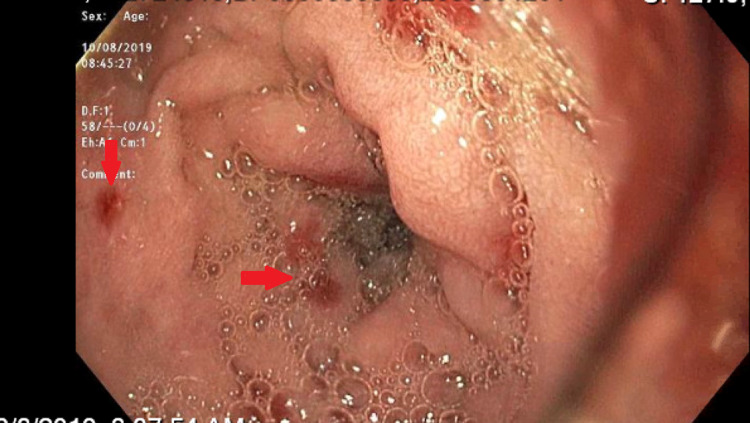
Endoscopic findings on upper endoscopy showing diffuse mucosal erythema, hemorrhagic erosions, and longitudinal ulcers

Gastroenterology was consulted for further evaluation of hematemesis. Upper endoscopy showed non-bleeding erosive gastropathy, duodenal erosions involving the duodenal bulb, and erosive mucosal changes (Figure 2). Rheumatology was also consulted for further evaluation of suspected IgA vasculitis. Skin biopsy of the left leg showed leukocytoclastic vasculitis with IgA and fibrin deposition within the walls of the superficial dermal vessels which confirmed the diagnosis of IgA vasculitis.

The patient was started on intravenous methylprednisolone 500 mg daily for a total of three days. He then received two methylprednisolone infusions in the outpatient setting followed by a steroid taper. Due to incomplete clinical response to steroids, mycophenolate mofetil 1000 mg twice daily was added to the treatment regimen and maintained for three months. His symptoms improved significantly, and he no longer complained of abdominal pain or diarrhea. The regimen was tapered off gradually, and he has since remained in remission.

## Discussion

IgA vasculitis is an inflammatory disorder characterized by IgA deposits on small blood vessels [1]. IgA vasculitis accounts for 45% of childhood vasculitis [4]; however, it is relatively rare in adults, with an incidence of five out of 100,000 patients in European studies [5]. Despite the benign course of the disease, adult-onset HSP has been associated with a more severe course that requires aggressive treatment [1]. The typical manifestations of HSP include palpable purpura (95%-100%), abdominal pain (35%-85%), and joint pain (60%-84%) [6]. Renal involvement has been reported in 20%-80% of cases, which manifests as microscopic or macroscopic hematuria, proteinuria, nephrotic syndrome, and/or acute kidney injury.

The GI manifestations of HSP result from submucosal hemorrhages, as well as fluid accumulation in the bowel wall from the underlying vasculitis and capillary hemorrhage. The small bowel (especially the second part of the duodenum) has been shown to be the most affected part of the GI tract. Stomach and colon involvement have also been reported [7]. Patients with GI involvement usually present with abdominal pain (85%), vomiting (40%), occult GI bleed (66%), massive lower GI bleed (20%), and diarrhea (20%) [8]. In rare cases, as in our case, GI manifestations were the initial presenting complaints that resulted in the diagnosis of HSP. Similar cases of GI bleed as the initial manifestation of HSP were reported by Hamazaoui et al. [9], Farah et al. [10], and Moles et al. [11]. GI manifestations were the presenting symptoms in 12% of HSP patients reported by Trapani et al. [12]. A summary of reported cases with treatment and outcome are compiled in Table 1. 

**Table 1:** Summary of the studies that reported Henoch-Schonlein purpura patients presenting with gastrointestinal manifestations Abbreviations: IgA, immunoglobulin A

Study	Year	Number of patients	Age (years)	Presenting symptoms	Associated symptoms	Definitive diagnosis	Treatment	Outcome
Hamzaoui et al. [9]	2011	3	20-60	Hematemesis and abdominal pain	Skin rash and arthritis	IgA vasculitis on skin biopsy	One case treated with methylprednisolone; the rest received symptomatic treatment	Complete recovery
Farah et al. [10]	2007	1	20	Abdominal pain	Skin rash and arthritis	IgA vasculitis on skin biopsy	Methylprednisolone and mycophenolate	Complete recovery
Moles et al. [11]	1991	1	20	Abdominal pain	Skin rash and arthritis	IgA vasculitis on skin biopsy	Methylprednisolone	Complete recovery
Trapani et al. [12]	2005	18	4-8	Abdominal pain	Skin rash and arthritis	IgA vasculitis on skin biopsy	Methylprednisolone	Complete recovery

Initial blood work in patients with HSP may show leukocytosis, elevated inflammatory markers, acute kidney injury, proteinuria, and/or hematuria. In patients with GI manifestations, abdominal CT may also show multifocal bowel wall thickening with skipped segments, with or without luminal narrowing, and nonspecific lymphadenopathy [13]. Patients with GI manifestations who undergo endoscopy characteristically have diffuse mucosal erythema, hemorrhagic erosions, petechia, and longitudinal ulcers especially in the second part of the duodenum [14]. However, skin biopsy remains the gold standard for the diagnosis. IgA deposition within the walls of the small blood vessels is pathognomonic for HSP. In 1990, the American College of Rheumatology proposed four diagnostic criteria for IgA vasculitis. The criteria include an age of less than or equal to 20 years at disease onset, palpable purpura, acute abdominal pain, and positive skin biopsy. The presence of at least two criteria confirms the diagnosis with 87.1% sensitivity and 87.7% specificity.

HSP is usually self-limited in 94% of children and 89% of adults. Symptomatic treatment with acetaminophen and/or nonsteroidal anti-inflammatory drugs is usually sufficient for skin rash and arthritis. However, patients with severe colicky abdominal pain, gastrointestinal, and renal involvement may benefit from steroid therapy. Oral prednisone can be started at 1 to 2 mg/kg daily for one to two weeks, followed by tapering dose at 0.5 mg/kg every day over the next week, then 0.5mg/kg every other day for one more week. Intravenous methylprednisolone is an alternative for patients who cannot tolerate oral intake. Mycophenolate mofetil (20-30 mg/kg twice daily) can be used in HSP patients with refractory GI involvement for one month, followed by a gradual taper over three months [15]. Refractory GI involvement is defined as the persistence of GI manifestations after three days of glucocorticoid treatment (at least 2 mg/kg/day) or relapse of these symptoms when glucocorticoids are tapered [16].

The efficacy of corticosteroid therapy in HSP patients with GI manifestations is still controversial. Many studies show a rapid improvement of GI symptoms within a few days of starting steroid therapy. Steroids may also prevent the recurrence of GI complications, including GI bleeding and intussusception [17]. On the other hand, few studies show no significant difference in the rate of gastrointestinal complications after steroid treatment. Huber et al. [18] and Ronkainen et al. [19] are both randomized control trials that studied the effect of corticosteroids on abdominal pain duration by using 14-day symptom diaries. The first study used 2 mg/kg per day of corticosteroids for one week, followed by tapering over the second week on a sample size of 40 children with HSP. The other study used 1 mg/kg per day of corticosteroids for two weeks, with weaning over three to four weeks on a total of 171 patients. Huber et al. did not find any difference in the median values of abdominal pain duration between the two groups after a one-year follow-up period (P= 0.8). In contrast, Ronkainen et al. found a mean reduction of 1.2 days of pain in patients who were treated with corticosteroids (P = 0.03).

A meta-analysis and systematic review included 15 articles that studied the effect of corticosteroids on the gastrointestinal manifestations of HSP [20]. The study showed that the early use of corticosteroids for children with HSP is associated with a statistically significant increase in the odds of abdominal pain resolution within 24 hours of starting treatment. However, this study was limited by the heterogeneity among the included studies. The source of heterogeneity could be attributed to the difference in sample size, the timing of corticosteroid administration after the disease onset, the dose of corticosteroids used, and the follow-up period among the studies. Larger randomized control trials and better-designed observational studies are needed for stronger evidence. This will help guide physicians in the early management of HSP with corticosteroids and improve the clinical outcomes for these patients.

## Conclusions

In conclusion, our case is one of the few cases that present adult-onset HSP. It is also unique in terms of presenting HSP as an unexpected cause of upper gastrointestinal bleed. Moreover, it supports the efficacy of corticosteroids in the management of gastrointestinal manifestations, as well as the effect of mycophenolate mofetil in the treatment of refractory cases.
